# Ki-67 as a controversial predictive and prognostic marker in breast cancer patients treated with neoadjuvant chemotherapy

**DOI:** 10.1186/s13000-017-0608-5

**Published:** 2017-02-21

**Authors:** Balázs Ács, Veronika Zámbó, Laura Vízkeleti, A. Marcell Szász, Lilla Madaras, Gyöngyvér Szentmártoni, Tímea Tőkés, Béla Á. Molnár, István Artúr Molnár, Stefan Vári-Kakas, Janina Kulka, Anna-Mária Tőkés

**Affiliations:** 1 2nd Department of Pathology, Semmelweis University, 1091 Budapest, Üllői út 93, Hungary; 2 MTA-SE-NAP Brain Metastasis Research Group, Semmelweis University, Budapest, Hungary; 3 MTA-TTK Lendület Cancer Biomarker Research Group, 1091 Budapest, Üllői út 93, Hungary; 4 Department of Clinical Oncology, Semmelweis University, 1083 Budapest, Tömő uta 25-29, Hungary; 5 1st Department of Surgery, Semmelweis University, 1082 Budapest Üllői út 78, Hungary; 6 University of Oradea, Romania, Faculty of Electrical Engineering and Information Technology, Str. Universitatii nr. 1, Oradea, Romania; 7 MTA-SE Tumor Progression Research Group, Semmelweis University, 2nd Department of Pathology, 1091 Budapest, Üllői út 93 Hungary

**Keywords:** Breast, Neoadjuvant chemotherapy, Ki-67, Predictive and prognostic markers

## Abstract

**Background:**

Studies have partly demonstrated the clinical validity of Ki-67 as a predictive marker in the neoadjuvant setting, but the question of the best cut-off points as well as the importance of this marker as a prognostic factor in partial responder/non-responder groups remains uncertain.

**Methods:**

One hundred twenty patients diagnosed with invasive breast cancer and treated with neoadjuvant chemotherapy (NAC) between 2002 and 2013 were retrospectively recruited to this study. The optimal cut-off value for Ki-67 labeling index (LI) to discriminate response to treatment was assessed by receiver operating characteristic (ROC) curve analysis. Kaplan-Meier curve estimation, log-rank test and cox regression analysis were carried out to reveal the association between Ki-67 categories and survival (DMFS = Distant metastases-free survival, OS = Overall survival).

**Results:**

Twenty three out of 120 patients (19.2%) achieved pathologic complete remission (pCR), whereas partial remission (pPR) and no response (pNR) to neoadjuvant chemotherapy (NAC) was detected in 60.8% and 20.0%, respectively. The distribution of subtypes showed a significant difference in pathological response groups (*p* < 0.001). Most of the TNBC cases were represented in pCR group.

The most relevant cut-off value for the Ki-67 distinguishing pCR from pNR cases was 20% (*p* = 0.002). No significant threshold for Ki-67 was found regarding DMFS (*p* = 0.208). Considering OS, the optimal cut-off point occurred at 15% Ki-67 (*p* = 0.006). The pPR group represented a significant Ki-67 threshold at 30% regarding OS (*p* = 0.001). Ki-67 and pPR subgroups were not significantly associated (*p* = 0.653). For prognosis prediction, Ki-67 at 30% cut-off value (*p* = 0.040) furthermore subtype (*p* = 0.037) as well as pathological response (*p* = 0.044) were suitable to separate patients into good and unfavorable prognosis cohorts regarding OS. However, in multivariate analyses, only Ki-67 at 30% threshold (*p* = 0.029), and subtype (*p* = 0.008) were independently linked to OS.

**Conclusions:**

NAC is more efficient in tumors with at least 20% Ki-67 LI. Both Ki-67 LI and subtype showed a significant association with pathological response. Ki-67 LI represented independent prognostic potential to OS in our neoadjuvant patient cohort, while pathological response did not. Additionally, our data also suggest that if a tumor is non-responder to NAC, increased Ki-67 is a poor prognostic marker.

**Electronic supplementary material:**

The online version of this article (doi:10.1186/s13000-017-0608-5) contains supplementary material, which is available to authorized users.

## Background

Breast cancer is the most common malignant tumor among women all around the industrialized world [1]. Proliferation is an essential characteristic of all cancer types, including breast cancer [2]. The proliferation activity of different tumors assessed with immunohistochemical detection of the cell-cycle specific antigen Ki-67 has been extensively studied in the last decade. Many studies have shown that Ki67 expression is a useful prognostic factor in breast cancer [3, 4]. Criscitiello et al. reported that Ki67 expression can identify a subset of patients among Luminal-B and node positive breast cancer cases who could benefit from addition of adjuvant chemotherapy to hormone therapy [5]. Contradicting this finding, Andre F. et al. reported that in the adjuvant setting Ki67 staining lacks analytical validity, moreover, no robust evidence indicates that Ki-67 staining predicts the efficacy of adjuvant chemotherapy [6]. Assessment of cellular proliferation by Ki-67 expression is not recommended in routine pathological evaluation by either International Ki-67 in Breast Cancer Working Group of the Breast International Group and North American Breast Cancer Group or the American Society of Clinical Oncology (ASCO) given the fact that it is not totally clear how Ki-67 measurements and thresholds could influence clinical decisions [7].

Nowadays neoadjuvant chemotherapy (NAC) has become an accepted therapy choice, even in early stage breast cancers. In the last few years several research studies have focused on the prognostic and predictive value of Ki-67 expression in neoadjuvant settings [8–10]. The International Ki-67 in Breast Cancer Group created guidelines for the assessment of Ki-67 with recommendations on pre-analytical and analytical procedures, as well as on interpretation, scoring, and data handling [11].

Achieving pathologic complete remission (pCR) remains a subject of controversy in terms of definition and its evaluation methods. The predictive value of pCR in relation to patient outcome in various breast cancer biological subtypes has been under debate. pCR has strongly predicted improved survival in triple negative breast cancer (TNBC) and HER2-enriched subgroups, while there are contradictory data related to the luminal subtypes [12, 13].

Even if significant efforts are currently made to select the group of patients who will benefit from NAC, there are no clear genomic markers that can predict the response rate [14]. There are contradictory data about Ki-67 as a predictive factor [8, 9] for response to NAC and there are several questions to be answered, such as the standardization of the methodology used for Ki-67 detection, or how Ki-67 expression in core biopsies is representative of the whole tumor. It is becoming clear that there is an urgent need for standardized Ki-67 diagnostics or a need for use of other markers associated with cell proliferation. Klauschen et al. reported that the computer assisted Ki-67 scoring approach correlated with the clinical endpoints [15]. Balmativola et al. found that 18% threshold of Ki-67 positive cells accurately discriminated the pCR and non-pCR patient groups [8]. In a recent study by Magbanua et al. it is nicely presented that in addition to single-gene studies, genomic approaches are needed to maximize the discovery of useful classifiers and druggable targets in the NAC setting [14].

The majority of previous research studies have focused mostly on the group of patients presenting pCR. Only a few studies have investigated the value of KI-67 in non-responders to NAC [8]. Even though partial responders to NAC represent the largest group with a very heterogeneous population, and although patients with minimal residual cancer burden after neoadjuvant chemotherapy often have the same excellent prognosis as patients who achieve pCR, there are very few studies offering additional information about the likelihood of partial response to NAC [16, 17]. Until this will happen, we need to investigate more clearly the relationship between pretherapeutic Ki-67 expression and response to neoadjuvant chemotherapy.

Our aim in this study was to find optimal cut-off values for Ki-67 expression that best correlates with response rates to NAC and with DMFS (distant metastasis-free survival) as well as with OS (overall survival). We also investigated the association between Ki-67, subtype and pathological response, as well as the prognostic potential of Ki-67 in partial responder patients. Furthermore, the prognosis prediction potential of Ki-67 was correlated to that of the conventional clinicopathological factors in multivariate analysis.

## Methods

A total of 120 patients diagnosed with invasive breast cancer and treated with NAC at Semmelweis University in Hungary between 2002 and 2013 were retrospectively recruited for this observational, cross-sectional cohort study. Patients were enrolled only if they had completed NAC, thereafter underwent surgery. The study was ethically approved by the Semmelweis University Institutional Review Board (SE-IKEB 120/2013).

The data recorded are partly presented in Table 1 and were as follows:

**Table 1:** Clinicopathological data of analyzed cases

		Number of cases	Total %	Valid %
Age	*40 ≥*	29	24.2	24.2
	*40 <*	91	75.8	75.8
cT	*T1*	19	15.8	15.8
	*T2*	73	60.8	60.8
	*T3*	15	12.5	12.5
	*T4*	13	10.8	10.8
pT	*pT0*	20	16.7	18.7
	*pT1*	40	33.3	37.4
	*pT2*	34	28.3	31.8
	*pT3*	9	7.5	8.4
	*pT4*	4	3.3	3.7
	*Unknown*	13	10.8	
cN	*N0*	46	38.3	40.4
	*N1*	55	45.8	48.3
	*N2*	9	7.5	7.9
	*N3*	4	3.3	3.5
	*Nx*	6	5.0	
pN	*pN0*	51	42.5	48.1
	*pN1*	36	30	33.9
	*pN2*	13	10.8	12.3
	*pN3*	6	5	5.7
	*Nx*	14	11.7	
Grade	*1*	1	0.8	0.9
	*2*	46	38.3	41.8
	*3*	63	52.5	57.3
	*Unknown*	10	8.3	
ER status	*Positive*	80	66.7	66.7
	*Negative*	40	33.3	33.3
PgR status	*20% >*	8	6.7	6.7
	*20% ≤*	49	40.8	41.2
	*Negative*	62	51.7	52.1
	*Unknown*	1	0.8	
HER2 status	*Positive*	41	34.2	34.2
	*Negative*	79	65.8	65.8
Histological type	*Lobular*	6	5.0	5.1
	*IBC NOS*	112	93.3	94.9
	*Other/Unknown*	2	1.7	
Molecular subtype	*Luminal A*	15	12.5	12.5
	*Luminal B/HER2-*	38	31.7	31.7
	*Luminal B/HER2+*	27	22.5	22.5
	*HER2+*	14	11.7	11.7
	*Triple-negative*	26	21.7	21.7
Response	*Complete*	23	19.2	19.2
	*Partial*	73	60.8	60.8
	*Non-responder*	24	20.0	20.0
Antracyclines	*Yes*	88	73.3	73.3
	*No*	32	26.7	26.7
Taxanes	*Yes*	99	82.5	82.5
	*No*	21	17.5	17.5
Platinum	*Yes*	31	25.83	25.83
	*No*	89	74.16	74.16
Trastuzumab	*Yes*	12	10.0	10.0
	*No*	108	90.0	90.0

- age at diagnosis, clinical and pathologic tumor size (cT and pT), nodal involvement (cN)
- in the pre-treatment biopsy: histological type and nuclear grade, Ki-67 LI, Estrogen Receptor (ER), Progesterone Receptor (PgR), and human epidermal growth factor receptor 2 (HER2) status (based on both immunostaining score and in situ hybridization analysis for HER2 score 2 cases)
- degree of response to therapy was categorized following Pinder et al. [18] in the histological sections of the post treatment surgical specimens as follows: Pathologic complete response (pCR) was defined as no residual invasive tumour and the absence of any residual invasive tumor in the lymph nodes. Partial response to therapy (pPR), either <10% of tumour remaining (pPRi), or 10–50% tumour remaining (pPRii), or >50% of tumor remaining but some evidence of response to therapy is present (pPRiii). Non responders (pNR) were defined as no evidence of response to therapy,
- Surrogate molecular subtypes were defined based on the 2013 St. Gallen Consensus Conference recommendations as Luminal-A, Luminal-B/HER2-, Luminal-B/HER2+, HER2+ and triple negative categories [19, 20].

### Immunohistochemical analysis

ER, PgR, HER2 status and Ki-67 LI were evaluated in the pre-treatment core biopsy specimens and in case of non pCR, on the surgical specimens as well, by immunohistochemistry (IHC). All immunohistochemical analyses were carried out at the 2nd Dept. of Pathology, Semmelweis University, Hungary with automated immunostainer system (Ventana Benchmark XT, Roche Diagnostics, Mannheim, Germany) according to the manufacturer’s instructions, using the following antibodies: 1:200 anti-ER (clone 6 F11), 1:200 anti-PgR (clone 312) and 1:150 anti-HER2 (clone CB11) antibodies purchased from Novocastra Laboratories Ltd (Newcastle upon Tyne, UK), and 1:100 anti-Ki67 (clone MIB1) antibody purchased from DAKO Gmbh (Hamburg, Germany). The antigen retrieval method was performed in the immunostainer with CC1 antigen retrieval solution on pH9 at 42 °C for 30 min.

The cut-off value for ER and PgR positivity was 1% positive tumor cells with nuclear staining. Hormone receptor (HR) negativity was defined as being negative for both ER and PgR. HER2 IHC positivity was defined as score 3+ complete, strong membrane staining in >10% of tumor cells. For IHC 2+ samples, fluorescent in situ hybridization (FISH) was performed to confirm gene amplification by using Zytovision ERBB2/CEN17 dual FISH probes. HER2 status was defined according to the ASCO/CAP guideline valid at the time of diagnosis (ASCO/CAP guideline 2007 and ASCO/CAP guideline 2013) [21, 22].

Ki-67 was scored as the percentage of positive tumor cell nuclei by counting a range of 400–500 cells (depending on the cellularity of the specimen), including also hot spot areas. The optimal cut-off value for Ki67 was defined by statistical analysis; however we also applied the 20 and 30% threshold values as mostly recommended cut-off scores in the literature.

#### Clinical outcome assessment

Overall survival was defined as the elapsed time from the date of diagnosis of the tumor by core biopsy to the date of death, or when patients were last censored if still alive. DMFS was defined as time from the date of primary diagnosis to the occurrence of first distant metastases. All patients were followed-up until the date of death or when censored at the date April 30, 2015 (median follow up time for OS and DMFS was 60.5 and 59 months, respectively).

#### Statistical analysis

All analyses were carried out using SPSS version 22.0 statistical software (SPSS Inc, Chicago, IL, USA). Differences in the distribution of characteristics between the parameters of patients with pCR or pPR and patients with pNR were evaluated using two-sided Fisher’s Exact Test. Two-sided Mann-Whitney-Wilcoxon test was used to define age distributions in pCR vs. pNR and vs. pPR. The optimal cut-off value for Ki-67 percentage to discriminate response to treatment was assessed by receiver operating characteristic (ROC) curve analysis. To identify the optimal Ki-67 threshold for NAC, only pCR and pNR cases were involved in ROC analyses, because pPR status is considered as a soft endpoint. Kaplan-Meier curve estimation, log-rank test and Cox regression analysis were carried out to analyze the association between Ki-67 categories and DMFS or OS. *P* values ≤0.05 were considered to be statistically significant.

## Results

### Baseline clinical and pathological data

Hundred and twenty patients met the inclusion criteria and their data were used in this study.

Mean patient age was 50.6 years (range, 29–74 years). The majority of patients (59.6%) had node-positive disease and cT2 tumors (60.8%). Tumors were ER-positive in 66.7% of cases and presented PgR positivity >20.0% in 41.2% of the analyzed samples. In 34.2% of cases HER2 positivity was detected. Of the 120 tumors, 12.5% were of Luminal-A, 31.7% of Luminal-B/HER2 negative, 22.5% of Luminal-B/HER2 positive, 11.7% of HER2+ and 21.7% of TNBC subtype (Table 1).

Twenty three out of 120 patients (19.2%) achieved pathologic complete remission (pCR), 73 (60.8%) showed partial remission (pPR), whereas no response to NAC (pNR) was detected in 24 cases (20.0%).

In the group of patients who obtained pPR, residual tumor was detected in lymph nodes only in 7 patients (9.6%), major response (>90% tumor regression) to NAC was observed in 8 cases (11.0%), a response rate between 50 and 90% was detected in 26 cases (35.6%), whereas a response rate <50% was observed in 32 cases (43.8%).

Non-responder patients (pNR, mean age: 56) showed significant difference (*p* = 0.008) in age compared to patients achieving pathologic complete response (pCR, mean age: 46). This association between pNR patients’ age and pCR patients’ age was mainly confined to the HR+ patient population.

Significant difference in HR status was observed between the pCR, pPR and pNR populations (*p* < 0.001): approximately three-quarters of the samples within the pCR group were HR negative as contrasted to the pPR and pNR groups, in which cases the proportion of HR+ samples was more than 3-times higher (Table 2).

**Table 2:** Association between age, hormone receptor positivity and therapy response

Therapy response category	N	HR status (%)		p^1^	Average age at diagnosis (year ± SD)			p^2^
		*HR+*	*HR−*		*HR+*	*HR−*	*all*	
pCR	23	5 (21.7)	18 (78.3)	<0.001	44.2 ± 12.6	46.4 ± 13.8	46.0 ± 13.3	0.008
pPR	73	55 (75.3)	18 (24.7)		50.0 ± 11.0	52.3 ± 12.8	50.4 ± 11.4	
pNR	24	20 (83.3)	4 (16.7)		55.9 ± 10.7	53.3 ± 14.5	55.6 ± 11.0	

HR – hormone receptor positive (+) or negative (−)

^1^two-sided Fisher’s exact test; ^2^Kruskal-Wallis H-test with Dunn’s posthoc (multiple comparison) test for all samples without regarding the HR status

### Defining cut-off points for Ki-67expression in the pCR and pNR groups

**Fig. 1:**
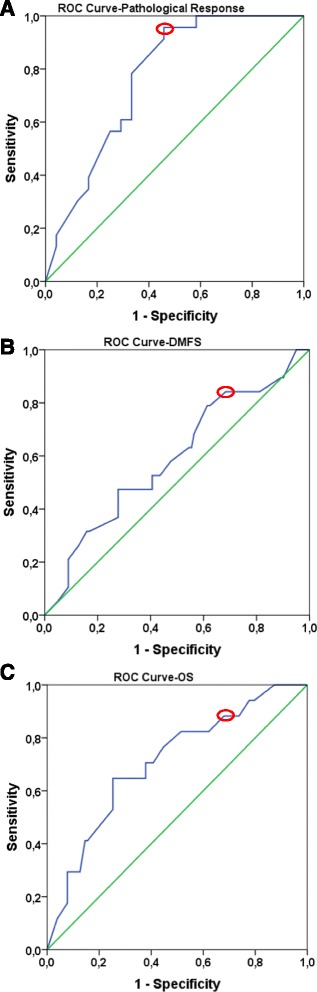
ROC curves to define optimal Ki-67 cut-off values for pathological response (**a**), DMFS (**b**), OS (**c**). *Green line* represents the diagonal reference line. *Blue line* corresponds to ROC curve. *Red circles* show the optimal cut-off values based on the ROC curves

ROC curve analysis was used in order to identify the optimal cut-off value of Ki-67 expression that could best predict response to NAC (Fig. 1a). The optimal Ki-67 cut-off value was 20% for distinguishing pCR from pNR patient cases (*n* = 47, AUC 0.767, sensitivity: 95.7%, specificity: 54.3%, *p* = 0.002). (Fig. 1a).

### Defining cut-off points for Ki-67 expression based on Survival (DMFS and OS)

We also investigated the optimal threshold values for Ki-67 regarding DMFS and OS. Based on DMFS, we were not able to detect a statistically significant cut-off value for Ki-67. The most relevant cut-off value was 20% (*n* = 120, AUC 0.591, sensitivity: 82.2%, specificity 35.7, *p* = 0.208) (Fig. 1b). On the basis of OS data, the optimal cut-off point occurred at 15% for Ki-67 (*n* = 120, AUC 0.708, sensitivity: 92.3%, specificity 29.6, *p* = 0.006) (Fig. 1c).

### Association between Ki-67, Subtype and Pathological Response

Pathological response and Ki-67 at investigated thresholds represented a significant association (Ki-67 15% *p* = 0.001, Ki-67 20% *p* = 0.010, Ki-67 30% *p* = 0.018). The proportion of Ki-67 low cases among non-responders was significantly higher compared to pPR and pCR cases (Additional file 1A). The distribution of subtypes showed a significant difference in pathological response groups (*p* < 0.001). Most of the TNBC cases were represented in pCR group, while luminal-A cases mainly occurred in pPR and pNR groups (Additional file 1B). The Ki-67 expression at any investigated cut-off points and subtypes also represented a significant correlation (*p* < 0.001 for all comparisons). Luminal-A subtype showed low Ki-67, while TNBC and HER2+ cases mostly had high Ki-67 (Additional file 1C). The association between Ki-67, subtype and pathological response was also investigated without Luminal-A cases, because NAC is not generally recommended in this subtype due to the high rate of pNR status in contrast with the favorable prognosis. Excluding Luminal-A cases, Ki-67 at any thresholds and pathological response did not show any significant association (Ki-67 15% *p* = 0.068, Ki-67 20% *p* = 0.122, Ki-67 30% *p* = 0.140) (Additional file 2A). Furthermore, Ki-67 expression at any investigated cut-off points also did not represent any significant linkage with subtypes (Ki-67 15% *p* = 0.410, Ki-67 20% *p* = 0.158, Ki-67 30% *p* = 0.173) (Additional file 2C). In contrast to this, subtypes were significantly linked to the pathological response groups (*p* < 0.001). The vast majority of Luminal-B cases were in pPR and pNR groups, while TNBC cases mostly occurred in pCR subgroup (Additional file 2B).

### Prognostic potential of Ki-67 status, Subtype and Pathological Response (DMFS and OS)

**Fig. 2:**
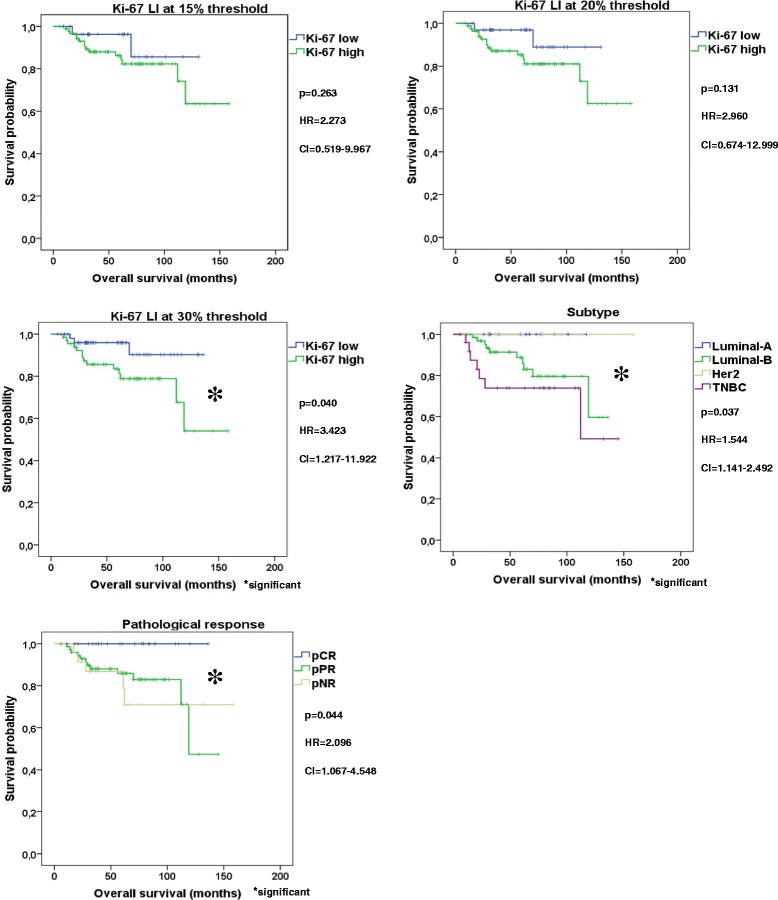
Kaplan Meier plots of Ki-67, subtype and pathological response. Ki-67 at 30% cut-off value furthermore subtype and pathological response were suitable to separate good and unfavorable patient cohorts regarding OS

Neither Ki-67 at any thresholds nor subtype and not even pathological response were suitable to distinguish patient cohorts with different DMFS (Ki-67 15% *p* = 0.391, Ki-67 20% *p* = 0.185, Ki-67 30% *p* = 0.566, subtype *p* = 0.771, pathological response *p* = 0.280). Regarding OS Ki-67 at 15% (*p* = 0.263) and at 20% threshold failed (*p* = 0.131), but Ki-67 at 30% cut-off value (*p* = 0.040) furthermore subtype (*p* = 0.037) as well as pathological response (*p* = 0.044) were suitable to separate patients into good and unfavorable prognosis cohorts (Fig. 2). When Luminal-A cases were excluded, neither Ki-67 at any cut-off points nor subtype not even pathological response were suitable to perform statistically significant splitting of our cohort into 2 patients’ group with different DMFS (Ki-67 15% *p* = 0.426, Ki-67 20% *p* = 0.179, Ki-67 30% *p* = 0.642, subtype *p* = 0.488, pathological response *p* = 0.222,) or with different OS (Ki-67 15% *p* = 0.975, Ki-67 20% *p* = 0.518, Ki-67 30% *p* = 0.158, subtype *p* = 0.072, pathological response *p* = 0.058, Additional file 3). We also investigated the utility of Ki-67 at 15, 20 and 30% thresholds as potential independent predictor of DMFS and OS adjusted by age, pathological response, hormone receptor status, subtypes, histological grade, lymph node, cT and pT status. Neither Ki-67 at any thresholds nor any other clinicopathological factors except pT status (*p* = 0.029) showed an independent association with DMFS (Table 3). However, Ki-67 at 30% threshold (*p* = 0.029) and subtype (*p* = 0.008) were independently linked to OS (Table 3). Without Luminal-A cases, Ki-67 at 30% cut-off point (*p* = 0.038) and subtype (*p* = 0.009) represented also an independent association with OS (Table 3).

**Table 3:** Multivariate Cox regression analysis of the Ki-67 and the clinicopathological factors regarding distant metastasis-free survival and overall survival

Prognostic factor	Multivariate Cox regression regarding distant metastasis-free survival (*n* = 120)			Multivariate Cox regression analysis regarding overall survival (*n* = 120)			Multivariate Cox regression without Luminal-A cases regarding distant metastasis-free survival (*n* = 105)			Multivariate Cox regression without Luminal-A cases regarding overall survival (*n* = 105)		
	HR	95% CI	*p*-value	HR	95% CI	*p*-value	HR	95% CI	*p*-value	HR	95% CI	*p*-value
Age	0.964	0.653–1.487	0.919	1.532	0.950–2.470	0.080	0.933	0.565–1.540	0.787	1.477	0.928–2.350	0.100
pT	2.397	1.094–5.252	0.029	1.931	0.914–4.082	0.085	1.812	0.914–3.592	0.086	1.903	0.895–4.046	0.079
cT	0.771	0.356–1.671	0.510	1.173	0.505–2.726	0.711	0.831	0.341–2.024	0.685	1.176	0.507–2.724	0.706
IHC Subtype	1.676	0.436–6.439	0.452	2.230	1.231–4.043	0.008	3.263	0.369–22.823	0.287	2.135	1.202–3.785	0.009
Histological grade	1.280	0.415–3.945	0.667	1.641	0.559–4.815	0.367	1.381	0.394–4.836	0.614	1.278	0.453–3.605	0.643
pN	1.146	0.681–1.929	0.607	1.478	0.881–2.479	0.139	1.334	0.765–2.325	0.309	1.417	0.874–2.296	0.157
cN	1.641	0.724–3.717	0.235	1.502	0.694–3.253	0.302	1.980	0.837–4.682	0.120	1.485	0.685–3.219	0.317
Hormone receptor status	0.905	0.061–13.456	0.942	0.561	0.201–1.565	0.270	2.833	0.244–20.808	0.623	0.784	0.289–2.130	0.633
Pathological response	0.851	0.263–2.749	0.787	1.056	0.376–2.965	0.918	0.814	0.217–3.049	0.760	1.899	0.300–2.694	0.850
Ki-67 LI_D15%	1.628	0.288–9.209	0.581	2.022	0.373–10.962	0.414	2.105	0.288–15.843	0.353	1.568	0.287–8.570	0.604
Ki-67 LI_D20%	2.575	0.511–12.962	0.251	2.847	0.528–15.341	0.223	3.864	0.495–28.809	0.175	2.338	0.434–12.604	0.323
Ki-67 LI_D30%	1.217	0.353–4.201	0.756	5.286	1.189–23.488	0.029	1.361	0.352–5.255	0.655	4.850	1.089–18.379	0.038

D15% = dichotomized at 15% threshold

D20% = dichotomized at 20% threshold

D30% = dichotomized at 30% threshold

### Ki-67 expression in the partial responder group

**Fig. 3:**
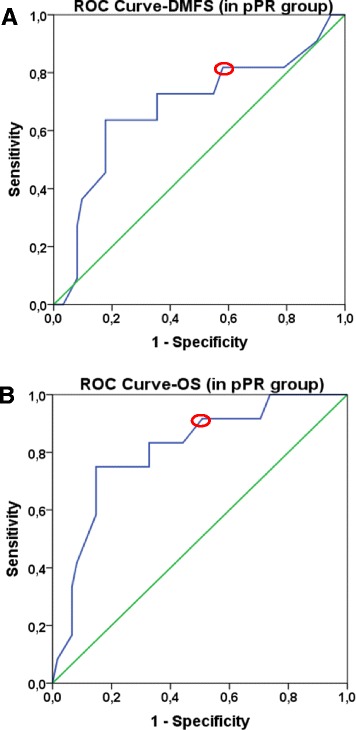
ROC curves to define optimal Ki-67 cut-off values for DMFS, OS in pPR group. *Green line* represents the diagonal reference line. *Blue line* corresponds to ROC curve. *Red circles* show the optimal cut-off values based on the ROC curves

The prognostic potential of Ki-67 was also investigated in pPR subgroup that represents a heterogeneous group with a response rate to NAC between 10 and 90%. Attempting to find the most relevant threshold for Ki-67, we could conclude that, the best cut-off value in pPR group based on DMFS was 20% (*n* = 73, AUC 0.683, sensitivity: 82.4%, specificity 41.5%, *p* = 0.055, Fig. 3a), and 30% based on OS (*n* = 73, AUC 0.808, sensitivity: 92.2%, specificity 52.6, *p* = 0.001, Fig. 3b). No significant association was found between Ki-67 and pPR subgroups (pPRi, pPRii, pPRiii; *p* = 0.653)

**Fig. 4:**
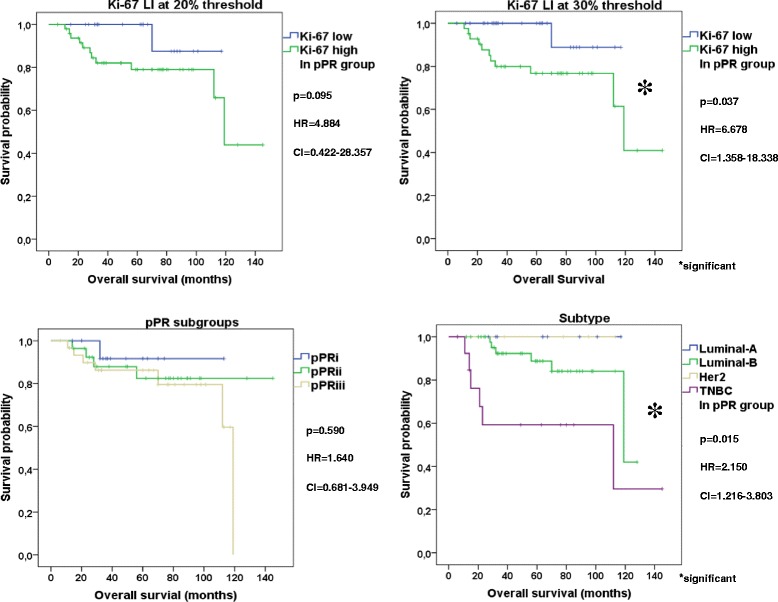
Kaplan Meier plots of Ki-67, subtype and pathological response in pPR group. Ki-67 at 20% threshold and pPR subgroups failed, while Ki-67 at 30% cut-off point and subtype were suitable to separate good and unfavorable patient cohorts regarding OS

For prognosis prediction, neither Ki-67 at any cut-off value (Ki-67 20% *p* = 0.233, Ki-67 30% *p* = 0.336), nor subtype (*p* = 0.218) not even pPR subgroups (*p* = 0.669) were able to distinguish patient cohorts with different DMFS. Regarding OS, pPR subgroups (*p* = 0.590) and Ki-67 at 20% threshold failed (*p* = 0.095), but Ki-67 at 30% cut-off point (*p* = 0.037) and subtype (0.015) were suitable to separate patients into good and unfavorable prognosis cohorts (Fig. 4).

## Discussion

Neoadjuvant systemic therapy is being increasingly used in the treatment of early stage breast cancer. Despite several classification systems developed for the assessment of pathologic response to NAC there is a current lack in uniformity regarding the definition of pathologic complete response [23, 24]. Since pCR is considered as the primary endpoint for response to chemotherapy, a majority of studies focus attention on pCR cases, while detailed analyses of partial responder or non-responder cases are relatively rare [8]. One of the hot topics in neoadjuvant therapy of breast cancer patients involves the question of reliable prognostic and predictive markers. A high number of studies have been conducted on the proliferation marker Ki-67, but due to the many controversies and debated issues this marker has not been fully integrated into clinical decision making. Some of the questions about the performance of Ki-67 as well as NAC in daily clinical practice concern the issue of cut points for Ki-67 and its use as prognostic or predictive marker. Different cut points are described, with values varying between 5 and 34% [25], between 3 and 94% for pCR, and between 6 and 46% for DMFS [10, 26]. The 2013 St. Gallen consensus recommended a Ki-67 cut-off value of 14% for the separation of luminal-A and B tumors, but in the footnote of the respective table there was a note indicating 20% as cut-off for “high” Ki67 LI. [19].

Our finding is in agreement with the results of Denkert et al. according to which Ki-67 is a mixed prognostic and predictive marker with its effect differing in opposite directions as regards prognosis and prediction [27].

Our study revealed that a Ki-67 cut-off value of about 20% distinguished pCR from pNR cases, whereas patients exhibiting Ki-67 expression lower than 30% demonstrated a higher chance of better overall survival. Increased Ki-67 LI was linked to worse OS, meaning that at least in some subgroups higher Ki-67 expression was related to increased response to NAC and was also associated with worse prognosis. These data may suggest that if a tumor belongs to the group showing no response to NAC, increased Ki-67 is a marker of poor prognosis.

Denkert et al. also suggest that based on Ki-67 expression there are three different groups of tumors, such as a group with low Ki-67 with good outcome, a group showing high Ki-67 and good outcome and a third group with high Ki-67 linked to poor outcome [27]. There are relatively few studies addressing the question of the role of Ki-67 in non-responder or pPR groups, even if the majority of cases treated with NAC show only partial response to chemotherapy.

In our study the majority of cases (60.83%) belonged to the pPR group. Based on Ki-67 expression, this group represented a mixture of tumors showing Ki-67 expression ranging from 1 to 100%. We analyzed whether the group of patients showing a near complete pathologic response (pPRi) showed higher Ki-67 expression compared to pPRii and pPRiii. According to our findings, there were no significant differences between these groups regarding Ki-67 expression. Based on the patients’ follow-up data and using ROC analysis, the most relevant prognostic cut-off value for the Ki-67 in the pPR group was 20% based on DMFS and 30% based on OS.

Balmativola et al. analyzed markers of non-response to NAC. Using ROC analysis, they identified a cut-off value of 18% for Ki-67 that performed well in differentiating the pNR and pCR + pPR categories [8].

In our study, Ki-67 expression was found to be higher than 20% in all patients achieving pCR and from the twenty-three pCR cases we detected distant metastases in one case only. In our study a Ki-67 LI of 20% was found capable of significantly distinguishing between the pCR and pNR groups. Despite this finding, however, it cannot be concluded that this is the only or best cut-off value for Ki-67, since the question then arises as to why a significant number of tumors with a Ki-67 value higher than 20% did not reach pCR.

Based on our results, both Ki-67 and subtype showed significant association with pathological response. However, when Luminal-A cases were excluded, only subtype and pathological response were significantly linked, so we could conclude that subtype has a significant impact on the association between Ki-67 LI and pathological response. In contrast to this, both Ki-67 and subtype were independently associated with OS, while pathological response did not show significant relation with OS. Furthermore, both Ki-67 and subtype were suitable to separate pPR patients into good and unfavorable prognosis cohorts.

## Conclusion

Neoadjuvant chemotherapy is more efficient in tumors presenting at least 20% Ki-67 expression. A cut-off value of 20% distinguished pCR from pNR cases. Increased Ki-67 LI was linked to worse OS, meaning that at least in some subgroups higher Ki-67 expression is related to increased response to NAC and is also associated with worse prognosis. Additionally, our data also suggest that if a tumor is non-responder to NAC, increased Ki-67 is a poor prognostic marker. Moreover, Ki-67 represented independent prognostic potential to OS, thus we can conclude that Ki-67 has potential utility in the clinical management of breast cancer. However, we can also state that Ki-67 in itself is not suitable to decide whether a breast cancer patient should be treated with NAC or not.

## Limitations

The weakness of our retrospective study is the relatively low number of cases, for which reason i.) we could not define the optimal Ki-67 cut-off point for each subtypes. ii.) We could not investigate whether Ki-67 is suitable to predict pathological response in each subtype. Similarly, we could not address the question of the prognostic potential of Ki-67 for each subtype in breast cancer patients who underwent neoadjuvant chemotherapy. iii.) We could not perform treatment-stratified analyses.

## Additional files

Additional file 1: Contingency tables of Ki-67 LI, subtype and pathological response. (DOC 90 kb)
Additional file 2: Contingency tables of Ki-67 LI, subtype and pathological response without Luminal-A cases. (DOC 87 kb)
Additional file 3: Kaplan Meier plots of Ki-67, subtype and pathological response without Luminal-A subtype. When Luminal-A cases were excluded, neither Ki-67 at any cut-off points nor subtype not even pathological response were suitable to perform statistically significant splitting of our cohort into 2 patients’ group with different OS. (DOC 305 kb)

## Abbreviations

ASCO: American society of clinical oncology
cN: Clinical lymph nodal involvement
cT: Clinical tumor size
DMFS: Distant metastasis-free survival
ER: Estrogen receptor
FISH: Fluorescent in situ hybridization
HER2: Human epidermal growth factor receptor 2
HR: Hormone receptor
IHC: Immunohistochemistry
NAC: Neoadjuvant chemotherapy
OS: Overall survival
pCR: Pathologic complete remission
PgR: Progesterone receptor
pNR: Non responders to neoadjuvant therapy
pPR: Pathologic partial remission
pT: Pathologic tumor size
ROC: Receiver operating characteristic
TNBC: Triple negative breast cancer
