# Supplementary material for: Ki-67 as a controversial predictive and prognostic marker in breast cancer patients treated with neoadjuvant chemotherapy
Source: Diagn Pathol. 2017 Feb 21;12:20. doi: 10.1186/s13000-017-0608-5 (PMC5320658; doi:10.1186/s13000-017-0608-5)
Supplement: Additional file 1: — Contingency tables of Ki-67 LI, subtype and pathological response. (DOC 90 kb) [file 13000_2017_608_MOESM1_ESM.doc]

| **Number of Cases**  **A**  Additional file 1. Contingency tables of Ki-67 LI, subtype and pathological response. | **Pathological Response** | | |  |
| --- | --- | --- | --- | --- |
|  | **pCR** | **pPR** | **pNR** | **Total** |
| **Ki-67 low (<15%)** | 0 | 19 | 10 | **29** |
| **Ki-67 high (≥15%)** | 23 | 54 | 14 | **91** |
| **Total** | **23** | **73** | **24** | **120** |
| **Number of Cases** | **Pathological Response** | | |  |
|  | **pCR** | **pPR** | **pNR** | **Total** |
| **Ki-67 low (<20%)** | 1 | 24 | 10 | **35** |
| **Ki-67 high (≥20%)** | 22 | 49 | 14 | **85** |
| **Total** | **23** | **73** | **24** | **120** |
| **Number of Cases** | **Pathological Response** | | |  |
|  | **pCR** | **pPR** | **pNR** | **Total** |
| **Ki-67 low (<30%)** | 6 | 31 | 16 | **53** |
| **Ki-67 high (≥30%)** | 17 | 42 | 8 | **67** |
| **Total** | **23** | **73** | **24** | **120** |

**B**

| **Number of Cases** | **Pathological Response** | | |  |
| --- | --- | --- | --- | --- |
|  | **pCR** | **pPR** | **pNR** | **Total** |
| **Luminal-A** | 0 | 9 | 6 | **15** |
| **Luminal-B** | 5 | 46 | 14 | **65** |
| **Her2** | 8 | 4 | 2 | **14** |
| **TNBC** | 10 | 14 | 2 | **26** |
| **Total** | **23** | **73** | **24** | **120** |

**C**

| **Number of Cases** | **Subtype** | | | |  |
| --- | --- | --- | --- | --- | --- |
|  | **Luminal-A** | **Luminal-B** | **Her2** | **TNBC** | **Total** |
| **Ki-67 low (<15%)** | 15 | 11 | 1 | 2 | **29** |
| **Ki-67 high (≥15%)** | 0 | 54 | 13 | 24 | **91** |
| **Total** | **15** | **65** | **14** | **26** | **120** |
| **Number of Cases** | **Subtype** | | | |  |
|  | **Luminal-A** | **Luminal-B** | **Her2** | **TNBC** | **Total** |
| **Ki-67 low (<20%)** | 15 | 16 | 2 | 2 | **35** |
| **Ki-67 high (≥20%)** | 0 | 49 | 12 | 24 | **85** |
| **Total** | **15** | **65** | **14** | **26** | **120** |
| **Number of Cases** | **Subtype** | | | |  |
|  | **Luminal-A** | **Luminal-B** | **Her2** | **TNBC** | **Total** |
| **Ki-67 low (<30%)** | 15 | 28 | 4 | 6 | **53** |
| **Ki-67 high (≥30%)** | 0 | 37 | 10 | 20 | **67** |
| **Total** | **15** | **65** | **14** | **26** | **120** |
