# Supplementary material for: Ki-67 as a controversial predictive and prognostic marker in breast cancer patients treated with neoadjuvant chemotherapy
Source: Diagn Pathol. 2017 Feb 21;12:20. doi: 10.1186/s13000-017-0608-5 (PMC5320658; doi:10.1186/s13000-017-0608-5)
Supplement: Additional file 2: — Contingency tables of Ki-67 LI, subtype and pathological response without Luminal-A cases. (DOC 87 kb) [file 13000_2017_608_MOESM2_ESM.doc]

| Additional file 2. Contingency tables of Ki-67 LI, subtype and pathological response without Luminal-A cases.  **A** | **Pathological Response** | | |  |
| --- | --- | --- | --- | --- |
|  | **pCR** | **pPR** | **pNR** | **Total** |
| **Ki-67 low (<15%)** | 0 | 10 | 4 | **14** |
| **Ki-67 high (≥15%)** | 23 | 54 | 14 | **91** |
| **Total** | **23** | **64** | **18** | **105** |
| **Number of Cases** | **Pathological Response** | | |  |
|  | **pCR** | **pPR** | **pNR** | **Total** |
| **Ki-67 low (<20%)** | 1 | 15 | 4 | **20** |
| **Ki-67 high (≥20%)** | 22 | 49 | 14 | **85** |
| **Total** | **23** | **64** | **18** | **105** |
| **Number of Cases** | **Pathological Response** | | |  |
|  | **pCR** | **pPR** | **pNR** | **Total** |
| **Ki-67 low (<30%)** | 6 | 22 | 10 | **38** |
| **Ki-67 high (≥30%)** | 17 | 42 | 8 | **67** |
| **Total** | **23** | **64** | **18** | **105** |

**B**

| **Number of Cases** | **Pathological Response** | | |  |
| --- | --- | --- | --- | --- |
|  | **pCR** | **pPR** | **pNR** | **Total** |
| **Luminal-B** | 5 | 46 | 14 | **65** |
| **Her2** | 8 | 4 | 2 | **14** |
| **TNBC** | 10 | 14 | 2 | **26** |
| **Total** | **23** | **64** | **18** | **105** |

**C**

| **Number of Cases** | **Subtype** | | |  |
| --- | --- | --- | --- | --- |
|  | **Luminal-B** | **Her2** | **TNBC** | **Total** |
| **Ki-67 low (<15%)** | 11 | 1 | 2 | **14** |
| **Ki-67 high (≥15%)** | 54 | 13 | 24 | **91** |
| **Total** | **65** | **14** | **26** | **105** |
| **Number of Cases** | **Subtype** | | |  |
|  | **Luminal-B** | **Her2** | **TNBC** | **Total** |
| **Ki-67 low (<20%)** | 16 | 2 | 2 | **20** |
| **Ki-67 high (≥20%)** | 49 | 12 | 24 | **85** |
| **Total** | **65** | **14** | **26** | **105** |
| **Number of Cases** | **Subtype** | | |  |
|  | **Luminal-B** | **Her2** | **TNBC** | **Total** |
| **Ki-67 low (<30%)** | 38 | 4 | 6 | **38** |
| **Ki-67 high (≥30%)** | 27 | 10 | 20 | **67** |
| **Total** | **65** | **14** | **36** | **105** |
