# Supplementary material for: Ki-67 as a controversial predictive and prognostic marker in breast cancer patients treated with neoadjuvant chemotherapy
Source: Diagn Pathol. 2017 Feb 21;12:20. doi: 10.1186/s13000-017-0608-5 (PMC5320658; doi:10.1186/s13000-017-0608-5)
Supplement: Additional file 3: — Kaplan Meier plots of Ki-67, subtype and pathological response without Luminal-A subtype. When Luminal-A cases were excluded, neither Ki-67 at any cut-off points nor subtype not even pathological response were suitable to perform statistically significant splitting of our cohort into 2 patients’ group with different OS. (DOC 305 kb) [file 13000_2017_608_MOESM3_ESM.doc]

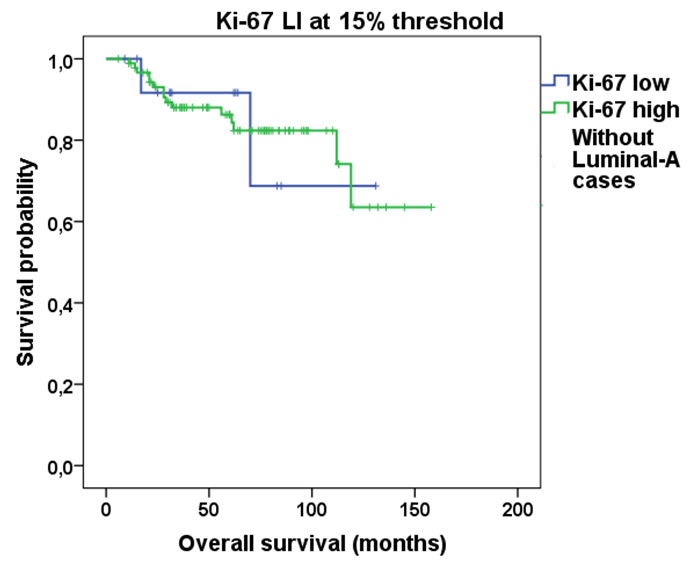

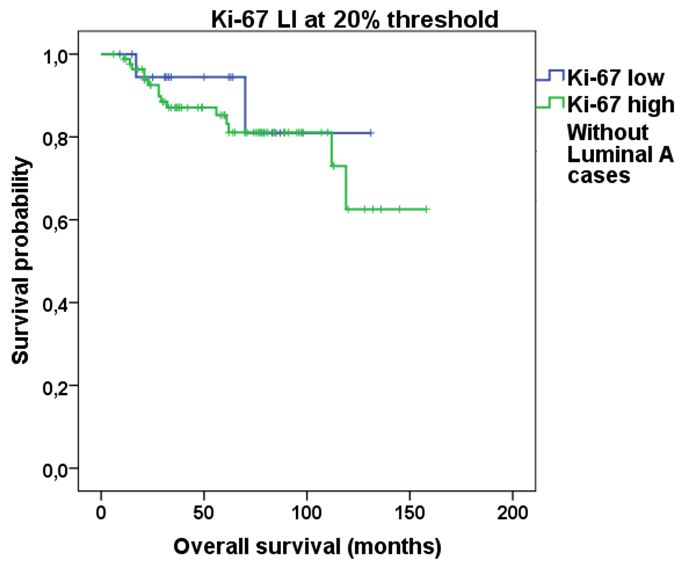

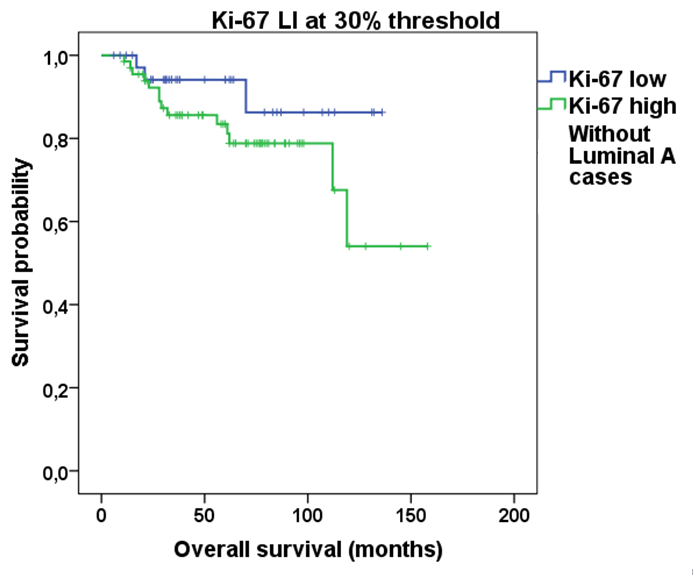

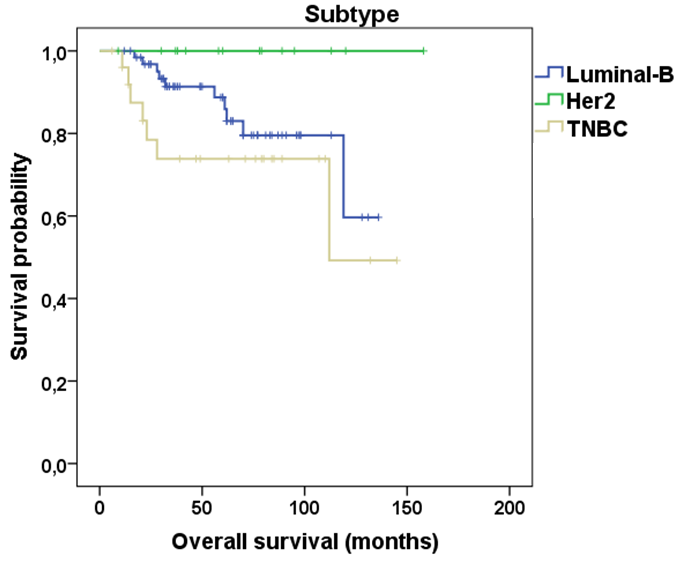

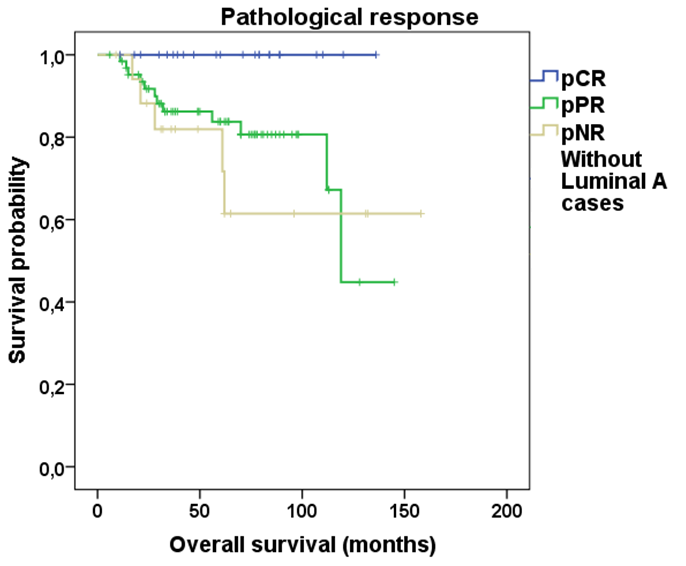


**p=0.158**

**HR=2.389**

**CI=0.686-8.323**

**p=0.072**

**HR=1.325**

**CI=0.777-2.262**

**Additional file 3.**  Kaplan Meier plots of Ki-67, subtype and pathological response without Luminal-A subtype. When Luminal-A cases were excluded, neither Ki-67 at any cut-off points nor subtype not even pathological response were suitable to perform statistically significant splitting of our cohort into 2 patients’ group with different OS.

**p=0.058**

**HR=2.339**

**CI=0.996-5.344**
